# Influence of iron ore properties on dioxin emissions during iron ore sintering

**DOI:** 10.1038/s41598-022-25752-8

**Published:** 2022-12-06

**Authors:** Xiaoteng Zhou, Vladimir Strezov, Tim Evans, Khushbu Salian, Mark Patrick Taylor

**Affiliations:** School of Natural Sciences, Faculty of Science and Engineering, Macquarie University, Sydney, NSW 2109 Australia

**Keywords:** Environmental chemistry, Chemical engineering

## Abstract

Iron ores are principal input materials for iron and steel-making industries. Quality of iron ores is one of the critical parameters for formation of environmental pollutants related to the steel-making process. Dioxins are identified as one of the most toxic pollutants emitted during ironmaking, specifically during the sintering process. This study applied four types of iron ores and analyzed their moisture, density, particle size distribution and element concentrations to investigate their effect on the dioxin formation during sintering. Each type of iron ore was processed in a sinter pot grate. During each processing route, exhausted dust and generated sinter products were collected and subjected to PCDD/F and PCB analysis. Statistical analysis was applied to assess correlations between properties of iron ores and exhausted dioxin emissions, identifying key contributors to dioxin formation during sintering process. Results showed that Fe in iron ores was positively and significantly related to PCB 114 formation in dust and confirmed its co-catalytic effect on dioxin formation. Concentrations of Al, Ti and Cl in iron ores greatly increased PCDD/F and PCB emissions in the sintered products compared to dioxins in dust samples. The S levels and density of iron ores were highly related to the increasing PCDD/F and PCB emissions in both sinter and dust samples. By contrast, concentrations of Si in iron ores played a significant role in decreasing PCDD/F and PCB emissions in both sinter and dust samples. This study also confirmed the optimum size (< 1 mm–2.59 mm) for iron ores, which helps reduce dioxin emissions without affecting the quality of iron and steel-making products.

## Introduction

Dioxins are highly toxic chemical substances classified under the Stockholm Convention as persistent organic pollutants. There are currently 419 types of identified dioxin-related compounds with 30 classified as highly toxic^1^. The primary class of dioxins are polychlorinated dibenzo para dioxins (PCDDs) and polychlorinated dibenzofurans (PCDFs). Additionally, the dioxin-like polychlorinated biphenyls (PCBs) are included in the dioxin family due to their similar toxic properties.

Evidence shows that PCDD/F and PCB exposure can cause reproductive disorders, skin lesions, developmental problems, impairment of the immune system and increased cancer risk^2^. The toxicity of the dioxin-related compounds is associated with their highly fat-soluble properties. As a result, they can easily accumulate in the human body and pose a significant health risk. Most importantly, estimates suggest that once they enter the human body dioxins can take between 7 and 11 years to reduce by half^1^.

Anthropogenic activities are the dominant source of dioxin-related pollutants, particularly industrial-chemical and combustion processes^3,4^. According to a multi-national inventory study, the iron and steel industry has been one of the largest contributors to dioxin emissions in Europe, Canada and Australia^5^. During the different iron and steelmaking processes, estimates show that approximately > 50% of the total dioxins are associated with the sintering processing^6^.

The sintering process is a pre-treatment step in the production of iron and steel. It is a thermal agglomeration process which blends iron ore fines, coke breeze and other additives, such as limestone, mill scale and recycled materials from downstream processes^7^.

In order to reduce PCDD/F and PCB emissions during the sintering process, three measures are widely used, including source control, process control and end-of-pipe treatments^6^. The use of inhibitors, such as N and S containing compounds, is a process control measure, but its efficiency is limited to 45–60% reduction in dioxin emissions^7^. In addition, as the N and S are introduced, the NH_3_ and SO_2_ emissions increased inevitably, posing a different environmental risk^6^.

The standard end-of-pipe treatments include electrostatic precipitator (with > 40% dioxin removal), activated carbon unit (> 90%), desulfurization system (> 50%) and wet scrubbing (> 60%)^8–10^. However, their efficiency of emission control is known to decrease with equipment age^11^.

The primary source control of dioxin formation is through avoidance of the dioxin formation precursors, such as Cu and Cl, in the raw materials, as Cu is known to act as a transition metal for catalysing reactions, while Cl is a key element for chlorination of the aromatic compounds^6,7^. Hence, the use of high-quality feedstock in sintering is a promising way to reduce dioxin emissions.

Previous studies investigated the influence of the quality of coke types and recycled fly ash on the dioxin formation^12–16^. However, considering iron ores are principal inputs for the sintering process, accounting for 90 wt.% of the sintering feedstock, the quality of the iron ores can significantly impact the PCDD/F and PCB emissions during sintering. A study by Cieplik et al.^17^ showed that the limonite generated higher PeCDD and HpCDF concentrations compared to magnetite. This indicates that iron ores with different physical and chemical properties play an important role in dioxin emissions during sintering. The aim of this study was to analyse the effect of element profiles and physical properties (i.e. moisture, density and fractions of particle sizes) of different iron ores on dioxin formation at a pilot plant scale sintering process. Exhaust dust and sintered products were collected during iron ore sintering and subjected to dioxin analysis. Statistical analysis was used to investigate correlations between properties of iron ores and dioxin emissions. Results from this study identified dioxin-related properties of iron ores, helping reduce dioxin emissions at source control during iron ore sintering.

## Methods

### Sample collection

Four types of iron ores, termed IO-1 to IO-4, were tested in this study. The iron ore samples were subjected to both physical and chemical analysis. The physical properties measured in this study include moisture, density and particle size analysis. Also, 18 elements were measured as chemical analysis for the four types of iron ores.

Each type of iron ore was mixed with the same raw materials of limestone, hydrated lime and coke. The quantities of input materials are detailed in Table S1. All input materials were mixed well prior to thermal processing in a sinter pot grate located in Outotec’s R&D centre in Germany. The scheme of the sinter pot grate and operating parameters are detailed in Figure S1. The sintering process for different iron ore types of IO-1, IO-2, IO-3 and IO-4 are designated as Route 1, Route 2, Route 3 and Route 4, respectively.

The sintered products generated from the sinter pot grate were collected after cooling and shattering. The dust particles were exhausted from the duct system of the sinter pot grate using an induced draft fan and were collected on filters. Both the sintered products and dust samples were subjected to dioxin analysis.

### Sample analysis

Iron ore samples were sieved using sieving stack, which returned particle size distribution in the range from 8 to 0.1 mm. The particle size distribution between 0.1 and 0.032 mm was determined by air screen sieving. Bulk density was the mean value of weighing a filled half litre cup twice, avoiding any compaction and using helium as displacement. The moisture was given in relation to the wet mass.

The element profiles were also analyzed for each type of iron ores. The elements of S and Cl were screened using Skyray Cube 100S XRF analyser, and other elements (Al, Ca, Cr, Cu, Fe, K, Mg, Mn, Ni, P, Pb, Si, Sn, Ti, V and Zn) were screened using Skyray Explorer 7000 XRF analyser. Multiple Certified Reference Materials (CRM) (e.g. CRM OREAS 192, 184 and 182) were applied to guarantee the quality assurance and quality control. The recovery rates for the 18 elements were 70–130%. The RSD values for all the element concentrations were < 3%.

The dioxins were analyzed at the National Measurement Institute in Sydney, Australia. For the analysis, fifteen ^13^C_12_ isotopically labelled PCDD/Fs and twelve ^13^C_12_ isotopically labelled PCBs (EPA1613-LCS and WP-LCS) were added into each sample. The samples were extracted by pressurised solvent extraction prior to concentration process. Further purification was performed using automated column chromatography (FMS Power-Prep, USA). The mono-ortho PCBs were eluted from the alumina column and analysed separately, while the non-ortho PCBs and PCDD/Fs were reverse eluted from the carbon column with toluene. Both extract portions were concentrated by vacuum concentration then solvent exchanged into dichloromethane and further evaporated by nitrogen blowdown in an amber gas chromatography vial with insert until just dry.

Two ^13^C_12_ isotopically-labelled PCDD/F and four ^13^C_12_ isotopically-labelled PCB recovery standards (EPA-1613ISS-STK and WP-ISS) were added to each vial prior to analysis by a high-resolution (> 10,000) magnetic sector mass spectrometer (Thermo Fisher DFS, Germany). The recovery rates for the individual surrogates ranged from 60 to 95% for PCDD/Fs and 59% to 125% for PCB congeners.

The data was then subjected to regression analysis to assess correlations between iron ore properties (density, moisture, particle sizes and element contents) and dioxin emissions (PCDD/Fs and PCBs) in exhausted dust samples and generated sinter products.

## Results

### Physical properties of iron ores

**Figure 1:**
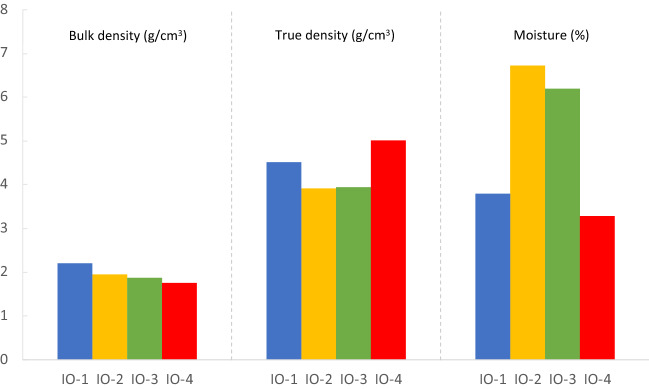
Bulk density (g/cm^3^), true density (g/cm^3^) and moisture (%) for the four types of iron ores used in this study.

The bulk density for the four iron ore types were found in the order of 1.75 g/cm^3^ (IO-4) to 2.2 g/cm^3^ (IO-1) (Fig. 1). While bulk density includes the volume of all pores within the samples, true density excludes the volume of the pores and voids. The true density of IO-4 presented the highest values of with 5.01 g/cm^3^ followed by IO-1 at 4.52 g/cm^3^ (Fig. 1).

The moisture of iron ores means the water content held in the iron ores of unit mass, which is a critical parameter for granule formation in a sintering process. IO-2 had the highest moisture values of 6.72%, while the lowest moisture was found in IO-4 sample (Fig. 1).

**Figure 2:**
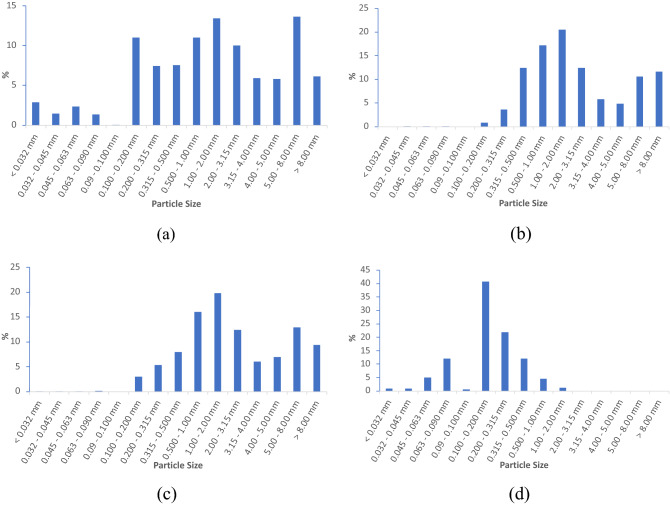
Particle size distributions for the four types of iron ores used in this study (**a**) IO-1, (**b**) IO-2, (**c**) IO-3, (**d**) IO-4.

The four iron ore samples presented different particle size behaviour (Fig. 2). The IO-1 sample had particle size distribution in both fine and coarse fractions (Fig. 2a). IO-2 and IO-3 had similar particle size distributions dominated by coarse fractions ranging from 0.315–3.15 mm to over 5 mm (Fig. 2b,c). By contrast, IO-4 primarily had small particles at sizes less than 1 mm (Fig. 2d).

### Chemical properties of iron ore samples

Table 1 presents the chemical analysis of the iron ore samples. The loss on ignition at 425 °C (LOI_425_) can be used as an indicator of the evolution of water from decomposition of the goethite fraction in the iron ore. IO-2 and IO-3 had the largest fraction of LOI_425_ at 9.4 and 9%, respectively. LOI_425_ for IO-1 was 4.6%, while IO-4 had no LOI_425_ indicating it was primarily comprised of hematite with 6.7% magnetite. The magnetite content for the other three iron ores was below detection limit. Loss on ignition at 1000 °C (LOI_1000_), which includes decomposition of both the goethite and the gangue material, was also the highest for IO-2 and IO-3 samples.

**Table 1:** Element concentrations and loss on ignition (LOI) in the four types of iron ores (IO) used in this study^*^.

Elements	Unit	IO-1	IO-2	IO-3	IO-4
Fe	%	62.0	57.5	56.7	66.5
Ca	%	0.04	0.05	0.09	0.22
Si	%	1.45	1.91	2.01	1.87
Mg	%	0.05	0.05	0.07	0.11
Al	%	0.53	0.40	0.69	0.04
Ti	%	0.06	0.05	0.07	0.03
Mn	%	0.12	< 0.05	< 0.05	0.09
P	%	0.09	0.05	< 0.03	< 0.03
Cl	%	0.016	0.004	0.014	0.002
S	%	0.015	0.008	0.013	0.008
LOI_425_	%	4.6	9.4	9	0
LOI_1000_	%	5.35	10.31	10.06	0.17
FeO	%	< 0.1	< 0.1	< 0.1	6.7

*Cr, Cu, K, Ni, Pb, Sn, V, Z were measured at below detection limit of 0.05% for all samples.

Eighteen elements were investigated for the four iron ore samples (Table 1), with their concentrations presented in %. Among the 18 elements, Fe was the predominant contributor with values from 56.7 to 66.5%. The element Si was the second contributor with values from 1.45 to 2.01% for the four iron ores. Metal elements (e.g. Ca, Mg, Al, Mn) presented relatively low concentrations in this study, and Cu had concentrations lower than the report limit. However, non-metals (i.e. Cl and S) were detected for the four iron ore samples, and both elements have been shown to influence the PCDD/F and PCB emissions during sintering^7^.

### Chemical analysis of the produced sinter

Table 2 presents the chemical analysis of the produced sinter. Only IO-3 showed Fe concentration below 57%. Alkali elements, S and Cl were all reduced in the final product. The basicity B2, a parameter influencing slag formation^18^ showed IO-1 had the highest basicity at 2.02, comparing to IO-3 with 1.69 showing the lowest basicity.

**Table 2:** Chemical analysis of the produced sinter expressed in weight %.^*^

Element	IO-1	IO-2	IO-3	IO-4
Fe (total)	58.7	57.8	56.3	59.4
Fe^2+^	3.7	3.8	3.7	5.7
CaO	8.5	9.4	9.8	9.1
SiO_2_	4.2	5.4	5.8	5
MgO	0.19	0.2	0.24	0.3
Al_2_O_3_	2.4	1.9	3.3	0.68
TiO_2_	0.18	0.14	0.17	0.07
Mn	0.17	< 0.05	0.07	0.09
P	0.09	0.05	0.04	< 0.03
B2 = CaO/SiO_2_	2.02	1.74	1.69	1.82

*K, BaO, Cu, Ni, Zn, Pb, Sn, V, Cr, S, Na, Cl were all measured below detection levels.

### Dioxin concentrations in exhausted dust samples

**Figure 3:**
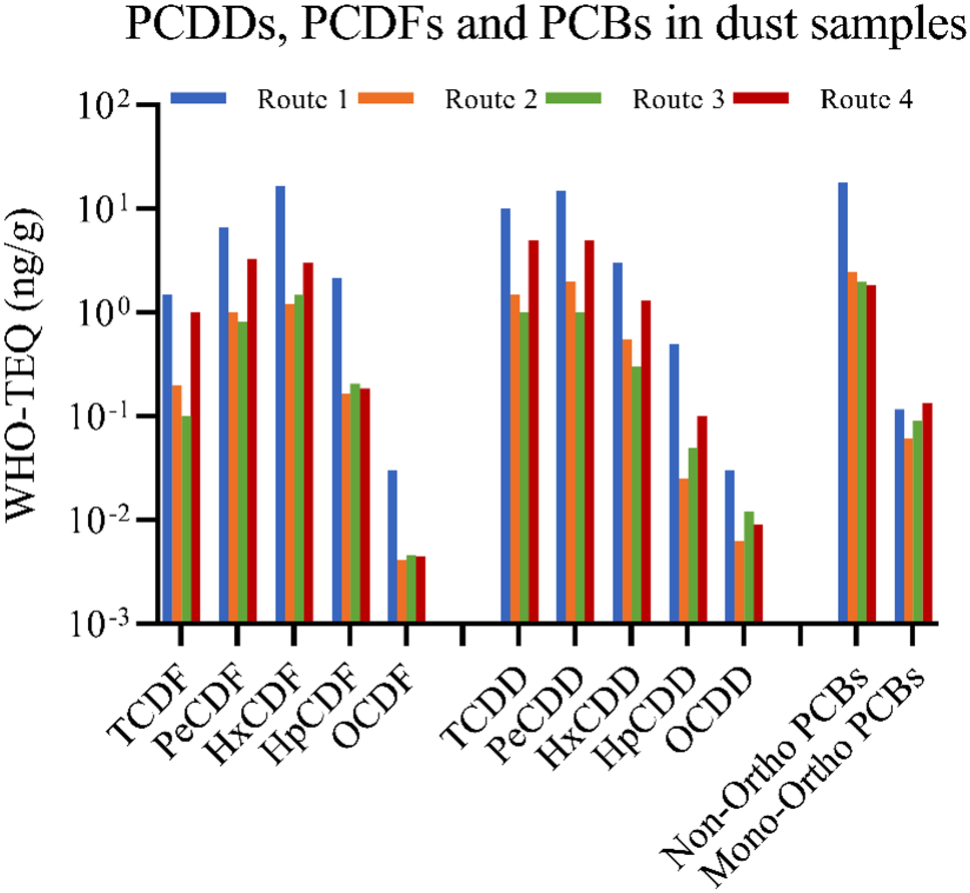
Concentrations of PCDDs, PCDFs and PCBs (WHO-THQ ng/g) in exhausted dust samples.

The route 1 using IO-1 presented the highest dioxin and PCB emissions compared to the other three processing routes (Fig. 3). The PeCDF and HxCDF were the dominant PCDF congeners among the four processing routes, while TCDD and PeCDD were the largest fractions in the PCDD emissions. The four processing routes all showed that the non-ortho PCBs were higher than mono-ortho PCBs (Fig. 3).

### Statistical results

The significant correlations between iron ore properties and PCDD/F homologues in dust are summarized in Table 3. Other significant correlations found between iron ore properties and PCB congeners are summarized in Table 4. The correlation results between iron ore properties and dioxins in the sintered products are summarized in Table S2.

**Table 3:** Statistical correlations between iron ore properties and dust-related dioxin emissions.

		TCDF (WHO-TEQ ng/g)	TCDD (WHO-TEQ ng/g)	PeCDF (WHO-TEQ ng/g)	PeCDD (WHO-TEQ ng/g)	HxCDF (WHO-TEQ ng/g)	HxCDD (WHO-TEQ ng/g)	HpCDF (WHO-TEQ ng/g)	HpCDD (WHO-TEQ ng/g)	OCDF (WHO-TEQ ng/g)	OCDD (WHO-TEQ ng/g)	Non-ortho PCB (WHO-TEQ ng/g)	Mono-ortho PCB (WHO-TEQ ng/g)
Si in iron ore (%)	r values	− 0.888	− 0.959*	− 0.958*	− 0.991**	− 0.981*	− 0.982*	− 0.968*	− 0.977*	− 0.969*	− 0.910	− 0.971*	− 0.413
	*p* values	0.112	**0.041**	**0.042**	**0.009**	**0.019**	**0.018**	**0.032**	**0.023**	**0.031**	0.090	**0.029**	0.587
Moisture in iron ore (%)	r values	− 0.891	− 0.790	− 0.788	− 0.670	− 0.561	− 0.726	− 0.471	− 0.586	− 0.476	− 0.481	− 0.446	− 0.962*
	*p* values	0.109	0.210	0.212	0.330	0.439	0.274	0.529	0.414	0.524	0.519	0.554	**0.038**
Iron ore particle size < 0.032 mm (%)	r values	0.947	0.991**	0.994**	0.995**	0.976*	0.995**	0.947	0.981*	0.949	0.926	0.939	0.600
	*p* values	0.053	**0.009**	**0.006**	**0.005**	**0.024**	**0.005**	0.053	**0.019**	0.051	0.074	0.061	0.400
Iron ore particle size < 0.045 mm (%)	r values	0.996**	0.989*	0.989*	0.948	0.891	0.969*	0.837	0.902	0.840	0.816	0.824	0.757
	*p* values	**0.004**	**0.011**	**0.011**	0.052	0.109	**0.031**	0.163	0.098	0.160	0.184	0.176	0.243

**Correlation is significant at the 0.01 level (2-tailed).

*Correlation is significant at the 0.05 level (2 tailed).

Significant values are in [bold].

**Table 4:** Statistical correlations between iron ore properties and concentrations of PCB congeners (WHO-TEQ ng/g) in dust samples.

		PCB 77	PCB 105	PCB 114	PCB 118	PCB 167
Fe in iron ore (%)	r values	0.907	0.901	0.967*	0.883	**− **0.086
	*p* values	0.093	0.099	**0.033**	0.117	0.914
S in iron ore (%)	r values	0.188	-0.125	0.032	0.134	0.972*
	*p* values	0.812	0.875	0.968	0.866	**0.028**
True density (g/cm^3^)	r values	0.924	0.929	0.984*	0.925	**− **0.022
	*p* values	0.076	0.071	**0.016**	0.075	0.978
Moisture (%)	r values	− 0.989*	**− **0.867	− 0.994**	**− **0.942	-0.260
	*p* values	**0.011**	0.133	**0.006**	0.058	0.740
Iron ore particle size < 0.063 mm (%)	r values	0.884	0.941	0.965*	0.911	**− **0.112
	*p* values	0.116	0.059	**0.035**	0.089	0.888
Iron ore particle size < 0.2 mm (%)	r values	0.752	0.954*	0.884	0.865	**− **0.306
	*p* values	0.248	**0.046**	0.116	0.135	0.694
Iron ore particle size < 0.315 mm (%)	r values	0.706	0.955*	0.853	0.852	-0.347
	*p* values	0.294	**0.045**	0.147	0.148	0.653
Iron ore particle size < 1 mm (%)	r values	**− **0.888	− 0.959*	− 0.971*	**− **0.936	0.078
	*p* values	0.112	**0.041**	**0.029**	0.064	0.922
Iron ore particle size < 2 mm (%)	r values	**− **0.830	− 0.955*	**− **0.935	**− **0.898	0.200
	*p* values	0.170	**0.045**	0.065	0.102	0.800
Iron ore particle size > 8 mm (%)	r values	**− **0.855	− 0.986*	− 0.955*	− 0.956*	0.081
	*p* values	0.145	**0.014**	**0.045**	**0.044**	0.919

**Correlation is significant at the 0.01 level (2-tailed).

*Correlation is significant at the 0.05 level (2 tailed).

Significant values are in [bold].

Results showed that concentrations of Si in the iron ore samples were significantly and negatively related to the PCDD/F and PCB levels in the exhausted dust samples (Table 3). The moisture of iron ores was also negatively related to the PCDD/F and PCB emissions (Table 3), and it was found to be significantly related to PCB 77 (r = − 0.989, *p* < 0.05) and PCB 114 (r = − 0.994, *p* < 0.001) in dust (Table 4).

The particle size at fine fractions (< 0.032 mm, < 0.045 mm, < 0.063 mm, < 0.2 mm and < 0.315 mm) were positively correlated to PCDD/F and PCB emissions (Tables 2, 3). However, the correlations presented negative values when the particle size increased to < 1 mm, < 2 mm and > 8 mm (Table 4).

## Discussion

Fe is a transition metal that can act as a catalyst to promote dioxin formation via the de novo synthesis^19,20^. Fe works with chlorides or chlorine to form carbonaceous and polycyclic aromatic structures leading to dioxin emissions^14,21^. However, a recent study showed that Fe in the iron ores had low catalytic activity during the de novo synthesis due to its oxide formation (e.g. Fe_2_O_3_)^22^. The study by Liu et al.^22^ found that the catalytic activity of Fe works with other transition metals, such as Cu, leading to high levels of dioxins, which means that Fe in iron ores is a co-catalyst during the sintering process. In this study, Cu concentrations were lower than the report limit for the four iron ore samples, and Fe was only positively related to PCB 114 at a significant level of *p* < 0.05 (Table 4). This also confirms that Fe in iron ores had limited catalytic action.

The presence of Cl in raw materials is another important factor for dioxin formation via de novo synthesis^23,24^. It is because Cl combines with metals, then metallic chlorides act as both catalysts and chlorinating agents to form dioxins during sintering^21,25^. The catalytic activity of metallic chlorides are ordered as CuCl_2_ > CuCl > (PbCl_2_, ZnCl_2_, FeCl_3_) > (SnCl_2_, CdCl_2_, NiCl_2_, FeCl_2_, MnCl_2_, CaCl_2_)^25^. Previous studies showed the addition of Cl contents can increase dioxin concentrations in exhausted fly ash samples^26,27^. In this study, the maximum Cl concentration was found in the IO-1 sample with 0.016% (Table 1), which exhibited the maximum PCDD/F congeners (Fig. 3). This study also found positive correlations at significant levels between Cl concentrations in raw materials and dioxins in the sintered products (Table S2), indicating the presence of Cl can not only increase dioxins in exhausted dust but also in the final sintering products.

Some studies suggest that SO_2_ can prevent PCDD/F formation^28–33^. There are two mechanisms proposed to explain the role of SO_2_ in reducing PCDD/F emissions^34^. One hypothesis is that the presence of SO_2_ consumes available Cl_2_ which is the major chlorinating agent in PCDD/F formation^29,35^. Another hypothesis is that SO_2_ can block metal catalysts (e.g. Fe and Cu) via converting metals into stable metal sulfides at even low temperature, as the catalytic effect of metal sulfide is lower than metal oxide (e.g. CuSO_4_ < CuO)^36,37^. In this study, there is no introduced SO_2_, but the four iron ores had S ranging from 0.008 to 0.015% (Table 1). The S concentrations in the iron ores were found to increase the concentrations of 1,2,3,4,7,8,9-HpCDF, PCB 77 and PCB 126 in the sintered products at a significant level of < 0.05 (Table S2). Also, a positive and significant correlation between S concentrations in the iron ores and PCB 167 in dust emissions was found in Table 4. Results from this study showed sulfur plays an important role in either decreasing or increasing dioxin emissions. Considering both reducing and oxidising conditions are present during sintering, the S content undergoes reactions with water and hydrogen before it is oxidised to SO_2_ and during these reaction steps can have different roles in the dioxin formation.

The other elements present in the iron ore also have influence on the dioxin formation mechanisms. Hinton and Lane^38^ found a negative correlation between Si in fly ash and emitted dioxin concentrations in MSW incinerator, but limited studies considered the role of Si on dioxin emissions. This study investigated the Si concentration in iron ores and the correlations between Si in raw materials and dioxin emissions. The Si concentrations in iron ores ranged from 1.45 to 2.01%, which was the second most abundant element in the iron ores (Table 1). This study also found that Si concentrations were negatively related to dioxin concentrations in both sintered products and dust samples at significant levels of < 0.01 and < 0.05 (Table 3 and Table S2). This is likely because during sintering, Si, in form of SiO_2_, strongly reacts with Fe oxides to form sinter melts, contributing to reduction of the available reactive iron, thereby inhibiting the iron chloride reactions as one of the catalytic steps to dioxin formation. SiO_2_ also contributes to lower basicity of the sinter. Iron ores producing IO-1 and IO-3 with higher basicity of the sinter (Table 2) clearly exhibited higher PCDD and PCDF concentrations (Fig. 3).

In addition to chemical properties, physical properties of bulk density, moisture and fractions of iron ore particle sizes are important parameters for iron ore quality^39,40^. For example, the high density with low porosity can slow down the flame front speed, impacting the combustion efficiency and the strength of the formed sinter^41^. This study showed that the high density of iron ores could contribute to the increased PCB 114 in dust samples and HxCDF in the formed sinter samples (Table 4 and Table S2).

The increasing moisture of iron ores can improve the strength of iron ore granules^42^. Previous study have found a clear negative correlation between moisture of iron ores and PCDD/F concentrations^43^. Although there were no significant correlations between moisture and PCDD/Fs found in this study, correlations between moisture and PCB 77 (r = − 0.989, *p* < 0.05) as well as PCB 114 (r = − 0.994, *p* < 0.01) were identified in Table 4. Results from this study showed that moisture of iron ores can decrease PCB emissions during the sintering process.

The fine particles of iron ores favour slag bonding in the sintering process^44^. Results from this study showed that high fractions of fine iron ores at < 0.032, < 0.045, < 0.063, < 0.2, and < 0.315 can significantly increase the PCDD/F and PCB emissions in the dust (Tables 3 and 4). With the increase in coarse iron ores, the fractions of particles at < 1 mm, < 2 mm and > 8 mm were negatively associated with PCBs (Table 4), indicating coarse iron ores can help reduce dioxin emissions. However, the excess of fractions in coarse iron ores (> 2.59 mm) can impact the melting properties and reduce the strength of granules^44^. Hence, the high fractions of iron ores at sizes from < 1 to 2.59 mm could achieve high sinter productivity, but at lower dioxin emissions.

## Conclusion

This study analyzed physical and chemical properties of four types of iron ores and investigated their corresponding dioxin emissions during pot grate sintering. The study confirmed the element concentrations in iron ores play an important role in influencing dioxin emissions. Specifically, Fe in iron ores acts as a co-catalyst, but Cl and S can dramatically increase dioxin concentrations in exhausted dust or generated sinter products. This indicates that iron ore fines released in fine particulate phase may have important role in dioxin formation in the off-gas, which needs further investigation. This study for the first time used statistical analysis to identify that Si in the iron ores has decreasing influence on the dioxin emissions. In addition to verifying the influence of density and moisture, this study identified the optimum size of iron ores (< 1–2.59 mm) when considering environmental impact and industrial productivity.

## Supplementary Information

Supplementary Information.

## Data Availability

The datasets used and/or analysed during the current study are available from the corresponding author on reasonable request.
