# Supplementary material for: Influence of iron ore properties on dioxin emissions during iron ore sintering
Source: Sci Rep. 2022 Dec 6;12:21080. doi: 10.1038/s41598-022-25752-8 (PMC9726927; doi:10.1038/s41598-022-25752-8)
Supplement: Supplementary file 1 — Supplementary Information. [file 41598_2022_25752_MOESM1_ESM.docx]

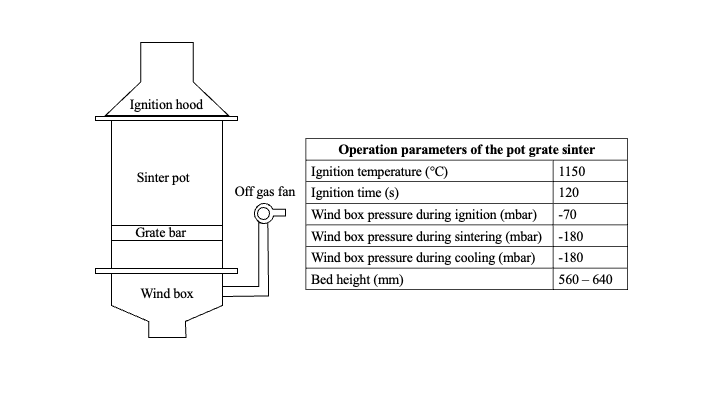


**Figure S1**. The scheme of the sinter pot grate and the operation parameters.

**Table S1**. The input materials in the sinter pot grate.

| **Materials** | **Amount supplied** |
| --- | --- |
| Iron ore 1 | 542 kg |
| Iron ore 2 | 542 kg |
| Iron ore 3 | 541 kg |
| Iron ore 4 | 580 kg |
| Limestone | 2 barrels |
| Hydrated lime* | Several kg |
| Coke | 1 barrel |

*Hydrated lime proportions for test 1, 2 and 3 using iron ore 1, 2, 3 accordingly were 0% (dry %), but its proportion for test 4 was 3% (dry %) to achieve the same sinter productivity and sinter strength in the agglomeration process with other three tests.

**Table S2.** Correlations between iron ore properties (element profiles, bulk density and particle sizes) and concentrations of PCDD/Fs and PCBs in sintered products.

|  |  | TCDD (WHO-TEQ ng/g) | 1,2,3,7,8-PeCDF (WHO-TEQ ng/g) | 1,2,3,7,8-PeCDD (WHO-TEQ ng/g) | 1,2,3,4,7,8-HxCDF (WHO-TEQ ng/g) | 2,3,4,6,7,8-HxCDF (WHO-TEQ ng/g) | 1,2,3,6,7,8-HxCDD (WHO-TEQ ng/g) | 1,2,3,4,7,8,9-HpCDF (WHO-TEQ ng/g) | PCB 77 (WHO-TEQ ng/g) | PCB 126(WHO-TEQ ng/g) | Non-ortho PCB (WHO-TEQ ng/g) |
| --- | --- | --- | --- | --- | --- | --- | --- | --- | --- | --- | --- |
| Si in iron ore (mg/kg) | r values | -.971^*^ | 0.144 | -.971^*^ | -0.926 | -0.926 | -.971^*^ | -0.733 | -0.362 | -0.733 | -0.564 |
|  | *p* values | 0.029 | 0.856 | 0.029 | 0.074 | 0.074 | 0.029 | 0.267 | 0.638 | 0.267 | 0.436 |
| Al in iron ore  (mg/kg) | r values | 0.277 | .979^*^ | 0.277 | 0.423 | 0.423 | 0.277 | 0.635 | 0.875 | 0.635 | 0.744 |
|  | *p* values | 0.723 | 0.021 | 0.723 | 0.577 | 0.577 | 0.723 | 0.365 | 0.125 | 0.365 | 0.256 |
| Ti in iron ore  (mg/kg) | r values | 0.293 | .970^*^ | 0.293 | 0.432 | 0.432 | 0.293 | 0.663 | 0.899 | 0.663 | 0.774 |
|  | *p* values | 0.707 | 0.030 | 0.707 | 0.568 | 0.568 | 0.707 | 0.337 | 0.101 | 0.337 | 0.226 |
| Cl in iron ore  (mg/kg) | r values | 0.664 | 0.714 | 0.664 | 0.736 | 0.736 | 0.664 | 0.942 | .993^**^ | 0.942 | .988^*^ |
|  | *p* values | 0.336 | 0.286 | 0.336 | 0.264 | 0.264 | 0.336 | 0.058 | 0.007 | 0.058 | 0.012 |
| S in iron ore  (mg/kg) | r values | 0.749 | 0.591 | 0.749 | 0.795 | 0.795 | 0.749 | .978^*^ | .965^*^ | .978^*^ | 1.000^**^ |
|  | *p* values | 0.251 | 0.409 | 0.251 | 0.205 | 0.205 | 0.251 | 0.022 | 0.035 | 0.022 | 0.000 |
| Bulk dentistry (kg/L) | r values | 0.902 | 0.367 | 0.902 | .957^*^ | .957^*^ | 0.902 | 0.809 | 0.611 | 0.809 | 0.700 |
|  | *p* values | 0.098 | 0.633 | 0.098 | 0.043 | 0.043 | 0.098 | 0.191 | 0.389 | 0.191 | 0.300 |
| Iron ore particle size < 0.5 mm (%) | r values | -0.624 | -0.645 | -0.624 | -0.675 | -0.675 | -0.624 | -0.927 | -.983^*^ | -0.927 | -.986^*^ |
|  | *p* values | 0.376 | 0.355 | 0.376 | 0.325 | 0.325 | 0.376 | 0.073 | 0.017 | 0.073 | 0.014 |
| Iron ore particle size < 5 mm (%) | r values | 0.312 | .966^*^ | 0.312 | 0.465 | 0.465 | 0.312 | 0.613 | 0.823 | 0.613 | 0.697 |
|  | *p* values | 0.688 | 0.034 | 0.688 | 0.535 | 0.535 | 0.688 | 0.387 | 0.177 | 0.387 | 0.303 |

** correlation is significant at the 0.01 level (2-tailed).

* correlation is significant at the 0.05 level (2 tailed).
